# Lower Urinary Tract Symptoms in Patients With Chronic Low Back Pain: A Cross-Sectional Study

**DOI:** 10.7759/cureus.45939

**Published:** 2023-09-25

**Authors:** Yasemin Yumusakhuylu, Hanife Caglar Yagci, Seyma N Bayindir

**Affiliations:** 1 Physical Medicine and Rehabilitation, Istanbul Medeniyet University, Istanbul, TUR; 2 Physical Medicine and Rehabilitation, Goztepe Prof. Dr. Suleyman Yalcin City Hospital, Istanbul, TUR

**Keywords:** uro-gynaecology, lumbar spine pain, urinary incontinence, lower urinary tract symproms, chronic low back pain

## Abstract

Introduction: This study aimed to determine the extent of lower urinary tract symptoms (LUTS) in patients with chronic low back pain (CLBP) and the relationship between LUTS and patients' clinical and functional factors.

Methods: Patients aged 40 to 80 who were admitted with CLBP were included. Demographic data and the duration of CLBP and LUTS were noted. Anteroposterior and lateral lumbar radiographs and lumbar MRI findings were recorded. Short Form 36 (SF-36) and the Oswestry Disability Index (ODI) were used for functional status assessment. For the LUTS evaluation, patients were asked to tick the symptoms from the list of LUTS prepared.

Results: We included 90 patients with CLBP. The frequency of urinary incontinence was 81.1%. The mean number of LUTS was 2.81±3.22. The LUTS rates were higher in patients with vertebral height loss (p = 0.03), with central (p = 0.02) and lateral spinal narrow canals (p = 0.03), and with facet hypertrophy (p = 0.04). The rates of LUTS were lower in patients with decreased lumbar lordosis (p = 0.02). The ODI and LUTS were found to be related (p = 0.01). The role limitations due to physical problems of the SF-36 subgroups and LUTS were significantly correlated (p = 0.01).

Conclusion: The incidence of the coexistence of CLBP and LUTS is high. Patients cannot match and report LUTS among their complaints, so physicians should inquire about LUTS in patients with CLBP and carry out the appropriate diagnosis and treatment.

## Introduction

Chronic low back pain (CLBP) is one of the most common musculoskeletal problems, and its frequency increases with age [1]. It is known that CLBP causes functional disability, falls, psychological problems such as depression and anxiety, sleep disorders, restricted social participation, and high socioeconomic costs [2]. Similarly, the prevalence of lower urinary tract symptoms (LUTS) increases with age, significantly affecting function and interfering with daily living activities [3]. The presence of LUTS deteriorates the quality of life and causes physical and emotional distress, social isolation, and a high financial burden [4]. Therefore, both health conditions have far-reaching effects on disability-adjusted quality of life [5].

Both conditions can be mistakenly accepted as a natural consequence of aging, and studies show that the incidence of coexistence is quite high [6]. Although hypothetical relationships have been suggested between CLBP and LUTS, the impact of non-specific CLBP on urinary function is not clear [7]. In the literature, the studies were mostly focused on urinary incontinence (UI) [8-10].

According to our clinical observations, CLBP was accompanied by other LUTS along with UI, regardless of age. The main purpose of our study was to determine the extent of LUTS in adults with chronic, non-specific LBP. Our secondary aim was to determine the relationship between these symptoms and the clinical and functional factors of our patient population.

## Materials and methods

Participants

Patients between 40 and 80 years of age who presented to Physical Medicine and Rehabilitation (PM&R) outpatient clinics with low back pain for at least six months were included in the study. To have a homogenous study group, patients who had previous low back or hip surgery, traumatic vertebral fractures, and diseases that would cause widespread chronic pain, such as fibromyalgia or inflammatory rheumatic disease, were excluded from the study. Those with known diseases related to the bladder, prostate, and urethra, such as chronic urinary tract infection, recurrent cystitis, urolithiasis, and benign prostatic hyperplasia, which may cause LUTS, were also excluded from the study. The first examination of the patient was performed by a physiatrist. Demographic data, the duration of LBP (in months), previous treatments, concomitant diseases, and LUTS such as UI, urgency, frequency, and dysuria were noted prospectively. Another physiatrist, unaware of the patient's clinical status, evaluated the anteroposterior and lateral lumbar radiographs. In radiological evaluation, osteophytes, loss of vertebral height, decrease in disc distance, and increase in sclerosis were recorded. In lumbar MRI, disc degeneration, facet joint hypertrophy, disc herniation, lateral and central spinal canal stenosis, and Modic changes, which are bone marrow lesions seen in a vertebral body on MRI, were recorded.

Measurements

Short Form 36 (SF-36) and the Oswestry Disability Index (ODI) are scales used for functional status assessment. The SF-36 questionnaire evaluates the last four weeks and consists of 36 items, while the fourth and fifth items are evaluated as yes/no; the other items are in triple and six-point Likert types. The scale consists of eight sub-parameters, which are physical functioning, role limitations due to physical health, role limitations due to emotional problems, energy and vitality, emotional well-being, social functioning, pain, and general health. Each item is scored between 0 and 100; low scores indicate poorness and high scores indicate well-being. The Turkish validity and reliability of the questionnaire were performed by Koçyiğit et al. [11].

The ODI was developed to assess pain-related disability in patients with low back pain. It includes 10 items that assess pain and daily living activities such as personal care, lifting, walking, sitting, standing, sleeping, sexuality, social life, and travel. Each item is measured on a 6-point ranking scale ranging from best to worst. Scoring is done on a scale of 0 to 5. Unmarked items are ignored. The highest score is 50, and the percentage value is calculated for the total score between 0 and 100. The Turkish validity and reliability of the index were performed by Yakut et al. [12,13]. For the LUTS evaluation, we asked the patients to tick the symptoms from the list of symptoms we prepared.

Statistical analysis

The results of our study were analyzed with SPSS Statistics version 25 (IBM Corp., Armonk, NY, USA). Descriptive analyses were given as percentage, mean, median, and standard deviation. Kruskall-Wallis and Mann-Whitney U test analyses were applied to examine the differences in the number of symptoms related to LBP-related disorders. Spearman correlation analysis and regression analysis were applied to examine the relationships between SF-36 quality of life and other measurement values and the number of LUTS. The Kolmogorov-Smirnov test was used to evaluate the fit of the data to a normal distribution. To compare categorical data, a Pearson chi-square test was used. Quantitative data with a normal distribution were analyzed with the Student's t-test, and data not conforming to a normal distribution were analyzed with the Mann-Whitney U test.

Ethical approval

The study was carried out per the Declaration of Helsinki and approved by the Ethics Committee of Istanbul Medeniyet University, Goztepe Prof. Dr. Suleyman Yalcin City Hospital (approval no. 2022/0062). A signed informed consent document was obtained from all patients.

## Results

After excluding patients who did not meet the criteria and did not want to participate, the study was completed with 90 patients with chronic mechanical LBP who presented to our outpatient clinics. The demographic and clinical characteristics of patients are summarized in Table 1.

**Table 1:** Demographic and clinical characteristics of patients enrolled in the study

Demographic & clinical characteristics		n	%
Gender	Male	21	23,3%
	Female	69	76,7%
Education	No education	6	6,7%
	Primary school	39	43,3%
	High school	22	24,4%
	University	23	25,6%
Occupation	None	54	60,0%
	Body	13	14,4%
	Office worker	23	25,6%
Comorbid diseases	None	57	63,3%
	One	26	28,9%
	More than one	7	7,8%
Smoking	-	69	76,7%
	+	21	23,3%

The mean duration of pain for the patients was 5.93±13.39 months, the visual analog scale (VAS) scores were 6.77±1.44, and ODI levels were 32.16±11.45. The physical examination findings of the patients are demonstrated in Table 2.

**Table 2:** Physical examination findings

Parameters	Results	n	%
Iliac crest sensitivity	-	27	30.0%
	+	63	70.0%
Great trochanter sensitivity	-	31	34.4%
	+	59	65.6%
Walking	Normal	80	88.9%
	Antalgic	10	11.1%
	Trendelenburg	0	0.0%
Lordosis	Normal	40	44.4%
	Decreased	42	46.7%
	Increased	8	8.9%
Lumbar sensitivity	-	16	17.8%
	+	74	82.2%
Straight leg raise	-	31	34.4%
	+	59	65.6%
Range of motion limitation	-	30	33.3%
	+	60	66.7%
Femoral stretching	-	50	55.6%
	+	40	44.4%
Deep tendon reflexes	-	84	93.3%
	+	6	6.7%
Loss of muscle strength	-	88	97.8%
	+	2	2.2%
Sensory loss	-	83	92.2%
	+	7	7.8%
Leg length inequality	-	88	97.8%
	+	2	2.2%
Hamstring shortening	-	34	37.8%
	+	56	62.2%

Around 73.3% of the patients had Modic changes, 96.7% had disc herniation, 12.2% had central spinal stenosis, 32.2% had lateral canal stenosis, and 38.9% had facet hypertrophy in the MRIs. About 81.1% of the patients had UI, 43.3% of the patients were diagnosed with stress urinary incontinence (SUI), 22.2% with urge urinary incontinence (UUI), and 15.6% with mixed urinary incontinence (MUI).

The average number of LUTS was 2.81±3.22. It was determined that the patients showed at least one and at most 10 LUTS among the 12 different symptoms questioned in the study. Around 56.7% of the patients had one of the questioned LUTS, which are summarized in Table 3.

**Table 3:** Lower urinary tract symptoms

Symptoms	Results	n	%
Nocturia	-	67	74,4%
	+	23	25,6%
Dysuria	-	73	81,1%
	+	17	18,9%
Frequency	-	59	65,6%
	+	31	34,4%
Difficulty starting urination	-	67	74,4%
	+	23	25,6%
Slowing of urine flow	-	66	73,3%
	+	24	26,7%
Dripping after urination	-	67	74,4%
	+	23	25,6%
Fullness in the bladder after urination	-	64	71,1%
	+	26	28,9%

The LUTS rate was not significantly associated with age, duration of pain, or VAS scores (p > 0.05). The LUTS rates were higher in patients with vertebral height loss (p = 0.03), in patients with central (p = 0.02) and lateral spinal stenosis (p = 0.03), and in patients with facet hypertrophy (p = 0.04). On the other hand, the rates of LUTS were lower in patients with decreased lumbar lordosis levels (p = 0.02). No correlation was found between LUTS frequency in patients with decreased disc distance, sclerosis, scoliosis, vacuum phenomenon, and MRI disc degeneration. The ODI and the number of LUTS shown by the patients were found to be positively, weakly, and significantly related (r = 0.26, p = 0.01). It can be said that the increase in the ODI level of the patients will also increase the number of LUTS.

It was determined that the role limitations due to physical problems of the SF-36 subgroups and the number of LUTS shown by the patients were negatively, weakly, and significantly correlated (r = -0.29, p = 0.01). It is seen that the increase in role limitations due to the emotional problems of the patients will decrease the number of LUTS. No significant relationship was found between other SF-36 subgroups and LUTS.

## Discussion

Urinary incontinence is a common clinical condition, and it has a widely ranging prevalence of 5% to 70%, with an average of 30% in various studies [14]. Particularly in the elderly, CLBP is another frequent disabling condition. The association between these two common conditions has frequently been mentioned in the literature. Large epidemiological studies demonstrated that the presence of one of these conditions seems to predispose them to the other [15].

In our study, the most common LUTS with LBP was UI, with a rate of 81.1%. In a study by Eliasson et al., 78% of the women with LBP reported UI, of whom 72% had SUI, 1% had UUI, and 27% had MUI [6]. Although the frequency of UI was similar, the rate of UI subtypes was different in our study. In our study, 53.45% of the patients were diagnosed with SUI, 27.4% with UUI, and 19.25% with MUI. Eisenstein et al. also drew attention to this issue and noticed a rare association between severe LBP and UUI that cannot be explained based on any conventional neurological or genitourinary pathology [7]. Similarly, Abreu et al. [16] and Kaptan et al. [17] reported no association between UI and LBP.

The prevalence of LUTS in adults has been reported between 20% and 64.3% in various studies [18,19]. Interestingly, in a study investigating LUTS in elderly patients with LBP, the prevalence was found to be 18% [5]. They attributed this result to the fact that the patients' LBP was acute; the answers were self-reported, and this issue was embarrassing for them. In the present study, 56.7% of patients with LBP had one LUTS. The high rate of LUTS in CLBP is a very important finding that reinforces the importance of addressing LUTS during the clinical evaluation of LBP so that appropriate therapy can be given to this patient group.

We did not detect a relationship between LUTS and the clinical parameters we evaluated at the examination, except for a decrease in lumbar lordosis. In a study investigating the relationship between spine curvatures and pelvic organ prolapses (POP), it was reported that a reduction in lumbar lordosis might play a role in POP [20]. The angle of the sacrum and tilt of the pelvis are related to the lumbar lordosis angle. It has been reported that in cases where lordosis decreases, the pelvis tilts posteriorly, and the pelvic floor muscles shorten and become more active [21]. Celenay et al. reported that UI was higher in the group with increased lumbar lordosis [22]. We would expect decreased pelvic floor muscle activity in this situation, and this is a known cause of SUI.

We could not find any study in the literature investigating the relationship between radiological imaging of the lumbar region and LUTS. Our study is unique in that it examines lumbosacral pathologies in detail with MRI and X-ray images and compares them with LUTS. According to the results of our study, the rate of LUTS was found to be higher in those with vertebral height loss (p = 0.03), those with central (p = 0.02) and lateral narrow spinal canals (p = 0.03), and those with facet hypertrophy (p = 0.04). This is an expected situation, as nerve roots can be affected by spinal canal stenosis. Kaptan et al. found no difference between lumbosacral pathologies and SUI in their study, but they stated that there might be a relationship between UUI in the presence of nerve root involvement, called radiculopathy [17].

In our study, we found that there is an increase in the number of LUTS with the increase in ODI scores. Similarly, Pavon et al. [23] reported that disability is one of the variables that affect the likelihood of UI in women with LBP. Another of our findings was that the increase in the number of LUTS was associated with the physical role limitations of SF-36.

Along with the positive aspects of our study, there were some limitations. The number of participants was sufficient for statistical calculations. Clinical examination, radiological findings, and LUTS interrogation were performed by three different investigators blinded to each other. In our study, MRI was not requested to avoid extra cost, but the fact that each patient had at least one MRI enriched our study. However, the fact that MRIs were taken in different centers was a limitation in terms of acquisition compatibility. Excluding patients with vertebral fractures from the study provided an advantage as well as a limitation. If we included these patients, we estimate that the frequency of LUTS would be much higher.

## Conclusions

Lower back pain is one of the most common problems encountered in PM&R outpatient clinics. Recently, UI and other LUTS have started to attract the attention of PM&R physicians. On the other hand, since both diseases are common conditions, their coexistence has recently been mentioned quite frequently. As we have shown in our study, the probability of LUTS accompanying LBP exceeded our expectations. Since patients cannot match this situation and report their complaints, physicians should inquire about LUTS in patients with LBP and carry out the appropriate diagnosis and treatment.
